# A Rare Cause of Reversible Splenial Lesion Syndrome: A Case Report with Epilepsy

**DOI:** 10.4274/balkanmedj.2017.0733

**Published:** 2018-01-20

**Authors:** Bilge Piri Çınar, Handan Akar, Abdullah Taylan

**Affiliations:** 1 Clinic of Neurology, Health Sciences University, Samsun Training and Research Hospital, Samsun, Turkey; 2 Clinic of Radiology, Health Sciences University, Samsun Training and Research Hospital, Samsun, Turkey

To the Editor,

Reversible splenial lesion syndrome can develop in association with various factors, such as infection, seizures and/or antiepileptic drug withdrawal (1,2,3). Complete resolution of magnetic resonance imaging findings in weeks or months is typical, irrespective of the clinical severity. Magnetic resonance imaging findings in Reversible splenial lesion syndrome include an oval or semi-oval signal increase in the splenium portion of the corpus callosum (boomerang sign) together with a reduction in apparent diffusion coefficient as evidence of cytotoxic oedema and diffusion restriction in the splenium on diffusion weighted imaging.

A 20-year-old male was admitted due to two episodes of loss of consciousness. No convulsion, vomiting or incontinence occurred in either of these two attacks. He reported that the last attack occurred after spending a long time on his computer, but he recalled no triggering event for the first attack, which took place two months previously. He remembered feeling uncomfortable and falling down while walking with a friend. He lost consciousness but returned to normal in a few minutes. His previous medical and family histories were normal. Physical, systemic and neurological examinations were normal. Laboratory tests including complete blood count, renal and hepatic function tests and electrolytes were within normal limits. Sharp wave activity was observed in the bilateral anterior temporal area at electroencephalography. An oval-shaped lesion was observed with T2 weighted imaging and fluid attenuated inversion recovery lining the midline of the corpus callosum splenium (mini-boomerang sign) (Figure 1). This lesion also showed evidence of restricted diffusion on diffusion weighted imaging.

The corpus callosum serves as a bridge permitting a constant exchange of information between the right and left hemispheres of the brain. Any injury can lead to various clinical disorders, such as cognitive impairment, urinary retention (4) and disconnection syndrome. The corpus callosum is resistant to ischaemia, but can be affected by factors such as seizures, metabolic changes, trauma, demyelinating disease, infection and alcohol use. In addition, the corpus callosum plays a role during generalised epileptic seizures. The splenium of the corpus callosum has a good blood supply and is rich in myelin content. Various theories, such as cytotoxic oedema, axonal damage, hyponatremia and oxidative stress have been proposed to explain the etiopathogenesis of Reversible splenial lesion syndrome, which is characterised by reversible splenium of the corpus callosum abnormalities (5,6,7,8).

In addition, increased white blood cells and interleukin levels in the cerebrospinal fluid have been reported in some cases (7,9).Whatever the underlying cause, when the underlying disease is corrected or, as in our case, when seizures are brought under control, this oedema improves spontaneously and the lesion disappears.

Oval-shaped hyperintensity in the splenium of the corpus callosum was observed at magnetic resonance imaging in our case, with no previous diagnosis of epilepsy, while diffusion restriction (mini-boomerang sign) was observed on diffusion weighted imaging accompanied by a decrease in apparent diffusion coefficient images (10). When the loss of consciousness and epileptiform abnormality determined at electroencephalography were evaluated together, epilepsy was suspected and the patient was started on antiepileptic therapy. Reversible splenial lesion syndrome cases with electroencephalography abnormalities and seizures have been reported previously (11). Intermyelin oedema can be responsible for lesions in the splenium of the corpus callosum during epileptic seizure or the postictal period. The lesion becomes invisible when cytotoxic oedema is resolved and the underlying cause is corrected. The previously observed splenial lesion was also no longer visible on contrast-enhanced magnetic resonance imaging one month after disease onset in our case. Reversible splenial lesion syndrome is rare but should be considered when lesions are detected in splenium of the corpus callosum in epileptic patients. Written informed consent was obtained from the patient.

## Figures and Tables

**FIG. 1:**
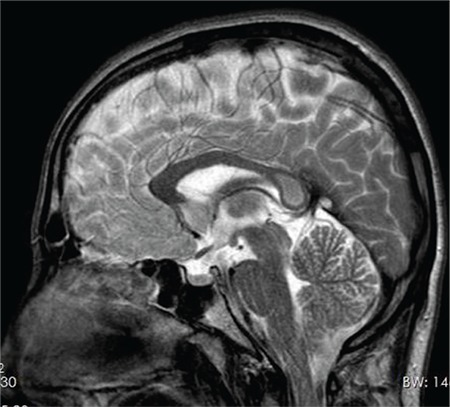
Hyperintensity in splenium of corpus callosum.
